# Supplementary material for: Multi‐dataset identification of innovative feature genes and molecular mechanisms in keratoconus
Source: J Cell Mol Med. 2024 Sep 19;28(18):e70079. doi: 10.1111/jcmm.70079 (PMC11412914; doi:10.1111/jcmm.70079)
Supplement: Supplementary file 2 — Material S2. [file JCMM-28-e70079-s002.zip › 7.GSEA analysis/1.ARL11/KEGG_LYSINE_DEGRADATION.html]

Details for gene set KEGG\_LYSINE\_DEGRADATION[GSEA]

|  || Dataset | ARL11.group.cls#h\_versus\_l |
| Phenotype | group.cls#h\_versus\_l |
| Upregulated in class | l |
| GeneSet | KEGG\_LYSINE\_DEGRADATION |
| Enrichment Score (ES) | -0.29073116 |
| Normalized Enrichment Score (NES) | -0.96295387 |
| Nominal p-value | 0.51417005 |
| FDR q-value | 1.0 |
| FWER p-Value | 1.0 |
Table: GSEA Results Summary

  

Fig 1: Enrichment plot: KEGG\_LYSINE\_DEGRADATION      
 Profile of the Running ES Score & Positions of GeneSet Members on the Rank Ordered List

  

| SYMBOL | TITLE | RANK IN GENE LIST | RANK METRIC SCORE | RUNNING ES | CORE ENRICHMENT || 1 | AASDH | na | 5325 | 0.157 | -0.0902 | No |
| 2 | SETMAR | na | 5544 | 0.150 | -0.0358 | No |
| 3 | ASH1L | na | 7594 | 0.100 | -0.0544 | No |
| 4 | BBOX1 | na | 9167 | 0.072 | -0.0705 | No |
| 5 | OGDHL | na | 10238 | 0.057 | -0.0783 | No |
| 6 | ALDH7A1 | na | 10913 | 0.048 | -0.0781 | No |
| 7 | SUV39H1 | na | 11531 | 0.041 | -0.0792 | No |
| 8 | EHMT1 | na | 12350 | 0.033 | -0.0895 | No |
| 9 | SETDB2 | na | 13082 | 0.026 | -0.0999 | No |
| 10 | ALDH3A2 | na | 13283 | 0.025 | -0.0958 | No |
| 11 | SETD1A | na | 14184 | 0.017 | -0.1147 | No |
| 12 | DLST | na | 14695 | 0.014 | -0.1239 | No |
| 13 | HADH | na | 15258 | 0.009 | -0.1363 | No |
| 14 | SETD1B | na | 15326 | 0.009 | -0.1347 | No |
| 15 | EHMT2 | na | 16133 | 0.003 | -0.1568 | No |
| 16 | AASDHPPT | na | 16416 | 0.001 | -0.1646 | No |
| 17 | TMLHE | na | 16646 | -0.001 | -0.1708 | No |
| 18 | SETD7 | na | 16806 | -0.002 | -0.1746 | No |
| 19 | ECHS1 | na | 17627 | -0.008 | -0.1951 | No |
| 20 | SETDB1 | na | 18784 | -0.016 | -0.2222 | No |
| 21 | OGDH | na | 19390 | -0.020 | -0.2317 | No |
| 22 | HADHA | na | 19491 | -0.020 | -0.2263 | No |
| 23 | ACAT2 | na | 20041 | -0.024 | -0.2325 | No |
| 24 | PLOD2 | na | 20729 | -0.029 | -0.2404 | No |
| 25 | NSD1 | na | 20801 | -0.030 | -0.2305 | No |
| 26 | DOT1L | na | 21125 | -0.032 | -0.2267 | No |
| 27 | PLOD3 | na | 22079 | -0.041 | -0.2377 | No |
| 28 | ACAT1 | na | 22983 | -0.050 | -0.2436 | No |
| 29 | GCDH | na | 24619 | -0.071 | -0.2620 | Yes |
| 30 | SETD2 | na | 24782 | -0.073 | -0.2370 | Yes |
| 31 | EHHADH | na | 24876 | -0.074 | -0.2096 | Yes |
| 32 | ALDH1B1 | na | 25126 | -0.078 | -0.1853 | Yes |
| 33 | ALDH9A1 | na | 25157 | -0.078 | -0.1546 | Yes |
| 34 | PIPOX | na | 25663 | -0.085 | -0.1347 | Yes |
| 35 | SUV39H2 | na | 27348 | -0.116 | -0.1364 | Yes |
| 36 | AADAT | na | 29654 | -0.177 | -0.1312 | Yes |
| 37 | PLOD1 | na | 30882 | -0.217 | -0.0789 | Yes |
| 38 | ALDH2 | na | 31150 | -0.226 | 0.0048 | Yes |
| 39 | AASS | na | 31583 | -0.243 | 0.0907 | Yes |
Table: GSEA details [plain text format]

  

Fig 2: KEGG\_LYSINE\_DEGRADATION      
 Blue-Pink O' Gram in the Space of the Analyzed GeneSet

  

Fig 3: KEGG\_LYSINE\_DEGRADATION: Random ES distribution      
 Gene set null distribution of ES for **KEGG\_LYSINE\_DEGRADATION**
